# Development and validation of a risk prediction model for hospital admission in COVID-19 patients presenting to primary care

**DOI:** 10.1080/13814788.2024.2339488

**Published:** 2024-04-29

**Authors:** Laure Wynants, Natascha JH. Broers, Tamara N. Platteel, Roderick P. Venekamp, Dennis G. Barten, Mathie PG. Leers, Theo JM. Verheij, Patricia M. Stassen, Jochen WL. Cals, Eefje GPM de Bont

**Affiliations:** a Department of Epidemiology, Care and Public Health Research Institute (CAPHRI), Maastricht University, Maastricht, The Netherlands; b Department of Development and Regeneration, KU Leuven, Leuven, Belgium; c Department of Family Medicine, Care and Public Health Research Institute (CAPHRI), Maastricht University, Maastricht, The Netherlands; d Julius Center for Health Sciences and Primary Care, University Medical Center Utrecht, Utrecht University, Utrecht, The Netherlands; e Department of Emergency Medicine, VieCuri Medical Center, Venlo, The Netherlands; f Dept. of Clinical Chemistry & Hematology, Zuyderland MC Sittard-Geleen/Heerlen, Heerlen, The Netherlands; g Department of Internal Medicine, School for Cardiovascular Diseases, CARIM, Maastricht University Medical Center, Maastricht, The Netherlands

**Keywords:** Covid-19, prognosis, clinical risk prediction model, general practice

## Abstract

**Background:**

There is a paucity of prognostic models for COVID-19 that are usable for in-office patient assessment in general practice (GP).

**Objectives:**

To develop and validate a risk prediction model for hospital admission with readily available predictors.

**Methods:**

A retrospective cohort study linking GP records from 8 COVID-19 centres and 55 general practices in the Netherlands to hospital admission records. The development cohort spanned March to June 2020, the validation cohort March to June 2021. The primary outcome was hospital admission within 14 days. We used geographic leave-region-out cross-validation in the development cohort and temporal validation in the validation cohort.

**Results:**

In the development cohort, 4,806 adult patients with COVID-19 consulted their GP (median age 56, 56% female); in the validation cohort 830 patients did (median age 56, 52% female). In the development and validation cohort respectively, 292 (6.1%) and 126 (15.2%) were admitted to the hospital within 14 days, respectively. A logistic regression model based on sex, smoking, symptoms, vital signs and comorbidities predicted hospital admission with a c-index of 0.84 (95% CI 0.83 to 0.86) at geographic cross-validation and 0.79 (95% CI 0.74 to 0.83) at temporal validation, and was reasonably well calibrated (intercept −0.08, 95% CI −0.98 to 0.52, slope 0.89, 95% CI 0.71 to 1.07 at geographic cross-validation and intercept 0.02, 95% CI −0.21 to 0.24, slope 0.82, 95% CI 0.64 to 1.00 at temporal validation).

**Conclusion:**

We derived a risk model using readily available variables at GP assessment to predict hospital admission for COVID-19. It performed accurately across regions and waves. Further validation on cohorts with acquired immunity and newer SARS-CoV-2 variants is recommended.

## KEY MESSAGES

A general practice prediction model based on signs and symptoms of COVID-19 patients reliably predicted hospitalisation.

The model performed well in second-wave data with other dominant variants and changed testing and vaccination policies.

In an emerging pandemic, GP data can be leveraged to develop prognostic models for decision support and to predict hospitalisation rates.

## Introduction

When the worldwide spread of SARS-CoV-2 started, many research initiatives emerged, almost exclusively in secondary care settings [1,2]. However, most patients initially contact their general practitioner (GP) and research initiatives in this specific setting could have the largest impact [3]. Risk assessment based on evaluating patient’s signs and symptoms can be complex and uncertain due to the highly variable clinical course of COVID-19 [4–6]. Multivariable prognostic models can help by providing patient-specific risks based on multiple predictors. Many prognostic models for COVID-19 exist but the vast majority are developed for prognostication after presentation in secondary care and of low quality [2]. The few models created on primary care data are not suitable to assist in prognostication following a physical assessment of symptomatic patients, as they do not take any symptoms or vital signs into account or are intended for remote assessment [7,11].

During the first COVID-19 wave, Dutch GP COVID-19 centres provided 24/7 care for their region [12]. During daytime hours, patients were triaged by regular practices, and those in which further physical assessment was needed were referred to the adjacent GP COVID-19 centre. During out-of-hours, the telephone team of the regular GP out-of-hours cooperative triaged patients and referred them to the COVID-19 location if deemed necessary. GPs in COVID-19 centres routinely assessed and recorded symptoms and vital parameters such as temperature, oxygen saturation, and respiratory rate. This provided a rich data source on the early symptoms and natural course of illness.

Despite high levels of protection within the community against SARS-CoV-2 due to acquired immunity, novel SARS-CoV-2 variants of concern continuously emerge and cause seasonal surges in hospital admissions. A model consisting of routinely available predictors that can accurately estimate hospitalisation risk due to COVID-19 after physical assessment by the GP may aid in selecting high-risk COVID-19 patients that might benefit most from early interventions aimed to reduce complications and prevent hospital admission. Integrating such models in routine care electronic health records systems would allow for continuous validation of model performance and recalibration of the model’s coefficients where needed by linking predicted risks based on GP data to hospital admission data.

This study aimed to develop a model to estimate 14-day hospital admission risk based on a combination of comorbidities, vital signs and symptoms of patients presenting with COVID-19 for a face-to-face consultation in general practice and to geographically cross-validate and temporally validate the model in a later wave.

## Methods

### Development and validation cohorts

The development database describes a cohort of adult patients presenting in eight Dutch GP COVID-19 centres from three regions (Western South Limburg, Eastern South and Central Limburg, and North Limburg) between 01/03/2020 and 31/05/2020, merged with the hospital admission records in the same regions until 15/06/2020. Inclusion criteria were face-to-face GP consultations with codes related to COVID-19, using the Dutch College of General Practitioners’ adaptation of ICPC (international classification of primary care) codes (see Tables S1–S3) [13]. Patients were excluded if a home visit to determine the death of a patient was not preceded by a COVID-19-related consultation in the study period. Other exclusion criteria were visits unrelated to COVID-19 complaints and home visits, resulting in patients deciding jointly with their GP not to visit the hospital due to a poor prognosis. Only the first visit for each patient is included in the current analysis.

The temporal validation database describes a cohort of adult patients presenting in 55 general practices in North Limburg between 13/03/2021 and 05/06/2021, merged with the hospital admission records from VieCuri (Venlo) until 19/06/2021. Only patients with confirmed COVID-19 were included; otherwise, in- and exclusion criteria were outlined above. Widespread testing and vaccination were available in the validation cohort and the dominant strains were alpha and delta, whereas it was the original (Wuhan) strain in the development cohort.

Patient data was pseudonymised after an independent third party hashed GP and hospital records and before collecting data in the electronic case report form.

### Outcomes

The primary outcome was hospital admission with COVID-19 within 14 days of the COVID-19-related consultation by the GP. The COVID-19 diagnosis was established according to local hospital protocol, which could include (repeated) SARS-CoV-2 RT-PCR testing and/or CoRADS-score 4 or 5 at pulmonary CT scans [14]. We also included GP-established (all-cause) mortality within 14 days but before hospital admission as an event in a composite outcome for the development and validation of the prognostic model as a safeguard against replicating potential inequities in case patients were denied access to hospital-based care at the peak of the pandemic, e.g. due to old age or comorbidity. For brevity, and because GP-established death within 14 days was infrequent, we will refer to the prediction of the composite endpoint as the prediction of hospital admission within 14 days. The secondary outcomes were hospital admission with COVID-19, ICU admission and in-hospital mortality at any time during the data collection period (minimum 14 days to maximum 106 and 98 days in the development and validation cohort, respectively). No blinding occurred but radiologists interpreting CT scans were unaware of GP findings per usual clinical practice. Those deciding on admissions were aware of GP findings per usual clinical practice.

### Predictors

This study used an electronic case report form in CASTOR EDC to extract data on repeated visits within the study period from GP databases, blinded for hospital data. The candidate predictors were selected based on the literature and prioritised based on frequency of occurrence [2,9,15], availability in general practice and subjective impression of importance among general practitioners (based on a poll at Maastricht University Primary Care Department). We considered age (in years), sex, overweight (BMI > 25, patient or physician reported), and current smoker as demographic and lifestyle predictors. Vital functions measured during the first GP visit included temperature, heart rate, respiratory rate, peripheral oxygen saturation, auscultation, duration of complaints, newly emerging confusion, chest pain or pressure, cough, sputum, haemoptysis, digestive complaints, fall event, dyspnoea, headache, and sudden worsening of complaints. The following comorbidities, events in the medical history, and use of drugs were candidate predictors: diabetes, history of cerebrovascular accident or transient ischaemic attack, chronic kidney disease, treatment for current malignancy, treatment for malignancy in the past, pre-existing hypertension, history of myocardial infarction or heart failure, chronic lung disease (incl. COPD and asthma), dementia, use of anticoagulants, use of immunosuppressives, and chronic use of corticosteroids.

### Statistical analysis

Sample size calculations assumed a hospital admission percentage of 5% at the start of the project, which was adjusted to 6.6% after data collection. Assuming a C-index of 0.80 and a shrinkage factor of 0.90, 4,790 patients would be needed to develop a model with the 44 degrees of freedom needed to test all candidate predictors and non-linear effects [16]. A priori, 1,000 patients for temporal validation would enable us to estimate the C-index with an error margin (confidence interval half-width) of 0.05. Based on this calculation, the validation cohort includes all GP visits during the peak of the second wave in North Limburg.

This study performed multiple imputations to handle missing predictor data (additional material S4) and modelled hospital admission using a logistic regression model built in the development cohort. Backward predictor selection (alpha 0.157) followed the a priori selection described above. Continuous predictors were modelled using restricted cubic splines with 3 knots to allow non-linear effects, and uniform shrinkage (based on the bootstrapped calibration slope) was applied.

The model was first evaluated in the first-wave development cohort using geographic leave-region-out cross-validation summarised with random effects meta-analysis [17,18]. Leaving out one region at a time for model development and evaluating the model in the left-out region, this procedure evaluates the model’s generalisability across regions. The developed prediction model (on all regions) was then assessed in the second-wave temporal validation cohort after filling in the predictor values in the prediction model equation. This evaluates the predictive performance after significant temporal changes occurred but is limited to North Limburg. In both cohorts, we assessed the C-index, calibration intercept, calibration slope and flexible (loess) calibration plots [17]. Pre-specified subgroup analyses were done based on patient sex and vaccination status.

We used the mice [19], RMS [20], psfmi and meta-packages in R [21,22].

### Ethics and open science

This study was approved by the ethical committee of the University Hospital Maastricht and Maastricht University) (METC azM/UM), the Netherlands and reported according to the TRIPOD guidelines [23]. The project metadata is publicly available at https://dataverse.nl/dataverse/pro-covid.

## Results

### Development cohort

**Figure 1.**
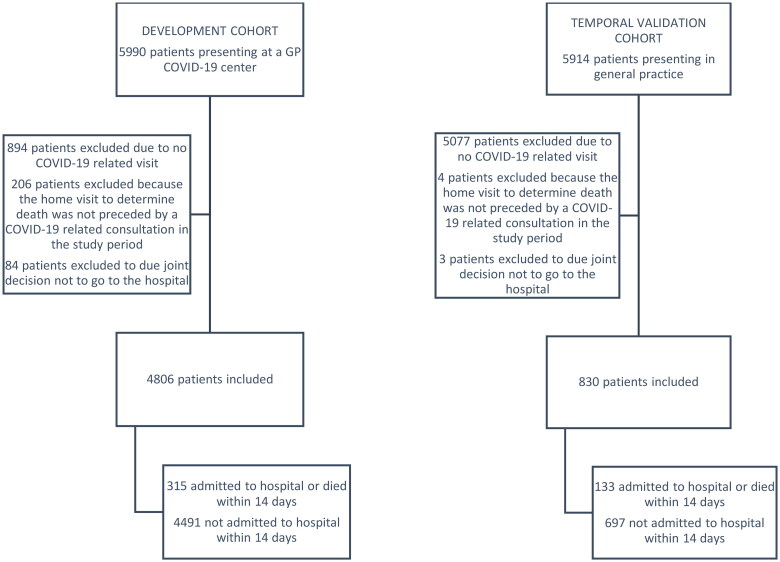
Flow chart of in- and exclusions.

A total of 4,806 patients were included in the development cohort, with a median age of 56 years and 56.4% female (Table 1a, Figure 1). Most patients presented with dyspnoea or cough; the median body temperature was 37.1 degrees Celsius (Table 1b). Common comorbidities were hypertension and chronic pulmonary disease (Table 1a). Within 14 days after the first GP visit, 292 patients (6.1%) were hospitalised, and 23 patients (0.5%) died at home (Table 2).

**Table 1a.** Patient demographics and clinical features are used for the development (*n* = 4,806) and validation (*n* = 830) cohort separately.

	Development cohort	Temporal validation cohort
Setting and location		
COVID-19 centre Western South Limburg	2022 (42.1%)	NA
COVID-19 centre Eastern South and Central Limburg	2006 (41.7%)	NA
COVID-19 centre North Limburg	778 (16.2%)	NA
Regular GP practices North Limburg	NA	713 (85.9%)
Out-of-hours centre North Limburg	NA	117 (14.0%)
Demographics		
Age (in years)	56 (18; 40; 70; 102)	56 (18; 45; 67; 95)
Sex, female	2710 (56.4%)	430 (51.8%)
Lifestyle and pre-existing comorbidity		
Overweight	214 (4.5%)	27 (3.3%)
Current smoker	342 (7.1%)	10 (1.2%)
Type I Diabetes	15 (0.3%)	3 (0.4%)
Type II Diabetes	309 (6.4%)	85 (10.2%)
Diabetes, type unknown	160 (3.3%)	14 (1.7%)
Insulin-dependent diabetes	193 (4%)	37 (4.5%)
Hypertension	1287 (26.8%)	248 (29.9%)
Cerebrovascular event	217 (4.5%)	16 (1.9%)
Heart disease	463 (9.6%)	44 (5.3%)
Cardiac arrhythmias	331 (6.9%)	21 (2.5%)
Any chronic pulmonary disease (incl. asthma and COPD)	1214 (25.3%)	143 (17.2%)
Asthma	533 (11.1%)	47 (5.7%)
COPD	534 (11.1%)	39 (4.7%)
Kidney disease	118 (2.5%)	10 (1.2%)
Current malignancy	81 (1.7%)	11 (1.3%)
Malignancy in past	210 (4.4%)	12 (1.4%)
Use of immunosuppressive agents	308 (6.4%)	37 (4.5%)
Chronic corticosteroid use	223 (4.6%)	47 (5.7%)
NSAID use	105 (2.2%)	37 (4.5%)
Anticoagulation	776 (16.1%)	153 (18.4%)
COVID-19 status		
Proven COVID-19^a^	122 (2.5%)	821 (98.9%)
Probable COVID-19^b^	4568 (95%)	9 (1.1%)
Contact with proven COVID-19 case	313 (6.5%)	333 (40.1%)
Contact with probable COVID-19 case	236 (4.9%)	440 (53.1%)

Reported statistics are median (min, first quartile, third quartile, max) or *n* (%).

^a^Proven COVID-19: confirmed COVID-19 according to a PCR or a rapid antigen test. Test availability was limited during the first wave in the Netherlands. ^b^Probable COVID-19: ICPC codes were R83.03 Sars-CoV-2 in addition to other ICPC codes that may indicate COVID-19 (see Table S1, Table S2, Table S3). In the second-wave validation cohort, patients were excluded if they did not have confirmed COVID-19 according to GP or hospital records.

**Table 1b.** Reported symptoms and vital signs were recorded during the first GP consultation, for the development (*n* = 4,806) and validation (*n* = 830) cohort separately.

	Development cohort	Temporal validation cohort
Symptoms reported during the first GP consultation		
Duration of symptoms (in days)	7 (0; 2.5; 14; 90)	7 (0; 4; 9; 35)
Sudden deterioration of symptoms	1340 (27.9%)	97 (11.7%)
Cough	3064 (63.8%)	544 (65.5%)
Sputum production	819 (17%)	119 (14.3%)
Haemoptysis	100 (2.1%)	23 (2.8%)
Dyspnoea	3095 (64.4%)	469 (56.5%)
Chest pain or pressure	1538 (32%)	152 (18.3%)
Sore throat	846 (17.6%)	93 (11.2%)
Loss of smell	259 (5.4%)	88 (10.6%)
Rhinitis	596 (12.4%)	94 (11.3%)
Deterioration of chronic illness symptoms (COPD, asthma, chronic cough)	598 (12.4%)	63 (7.6%)
Myalgia	590 (12.3%)	205 (24.7%)
Fatigue	1376 (28.6%)	333 (40.1%)
Shivering	448 (9.3%)	74 (8.9%)
Headache	876 (18.2%)	163 (19.6%)
Diarrhoea	469 (9.8%)	89 (10.7%)
Other abdominal complaints	1272 (26.5%)	300 (36.1%)
Confusion	159 (3.3%)	17 (2%)
Falling incident	77 (1.6%)	25 (3%)
Vital signs measured at the first GP consultation		
Auscultation abnormalities	1145 (23.8%)	218 (26.3%)
Auscultation abnormalities one-sided	495 (10.3%)	106 (12.8%)
Auscultation abnormalities two-sided	650 (13.5%)	112 (13.5%)
Crepitations	592 (12.3%)	158 (19%)
Respiratory rate	18 (5; 15; 24; 40)	20 (10; 16; 30; 40)
Diastolic blood pressure	80 (38; 71; 90; 140)	80 (40; 70; 84; 110)
Systolic blood pressure	132 (80; 120; 148; 220)	127 (80; 118; 140; 180)
Heart rate	86 (30; 76; 100; 194)	87 (52; 78; 99; 150)
Body temperature	37.1 (34; 36.7; 37.6; 40.9)	37.4 (35.2; 37; 38; 40.8)
Peripheral oxygen saturation	98 (40; 95; 98; 100)	97 (56; 94; 98; 100)

Reported statistics are median (min, first quartile, third quartile, max) or *n* (%).

**Table 2.** Outcomes for the development (*n* = 4,806) and validation (*n* = 830) cohort separately.

	Development cohort	Temporal validation cohort
Hospital admission within 14 days	292 (6.1%)	126 (15.2%)
Pronounced dead by GP within 14 days (before hospital admission)	23 (0.5%)	7 (0.8%)
Hospital admission at any time during follow-up^a^	294 (6.1%)	126 (15.2%)
ICU admission (any time)^b^	60 (1.2%)	24 (2.9%)
In-hospital mortality (any time)^c^	43 (0.9%)	16 (1.9%)

Reported statistics are median (min, first quartile, third quartile, max) or n (%).

^a^Hospital follow-up was obtained 2 weeks after the last inclusion of patients visiting general practice, and hence varied between patients from 2 weeks to 3.5 months.^b^Among admitted patients, we do not have complete follow-up on ICU admission of 1 (0.02%) and 18 (2.2%) patients of the development and validation cohort, respectively, because they were transferred to another hospital before any ICU admission took place. Consequently, ICU admission frequency may be underestimated.^c^Among admitted patients, we do not have a complete follow-up on in-hospital mortality of 9 (0.2%) and 25 (3.0%) patients of the development and validation cohort, respectively, because they were transferred to another hospital (before or after any potential ICU admission). Consequently, in-hospital mortality frequency may be underestimated.

The multivariable prediction model fitted on the development data showed that being male, auscultation abnormalities, confusion, cough, haemoptysis, digestive complaints, headache, chronic kidney disease, lower oxygen saturation and higher body temperature increased the probability of hospital admission (Box 1, Table S5). Smoking, chest pain/discomfort, sputum production, current or past treatment for malignancy, and COPD decreased the probability. The probability of hospital admission within 14 days peaks for 60-year-olds and around seven days of complaint (Figure S6). Only 1.7% of patients had predicted admission risks > 50% (Figure S7). The model could distinguish between patients who do and do not get admitted within 14 days in all three regions, with a leave-region-out cross-validated pooled C-index of 0.84 (Table 3, Figure S8). The predicted risks corresponded well to observed admission risks in each region and sex subgroups (Table 3, Figure S7).

**Table 3.** Model performance (leave-region-out cross-validation and temporal validation).

	Leave-region-out cross-validation	Temporal validation
C-index^a^	0.84 (95% CI 0.83 to 0.86) (95% PrI 0.78 to 0.89)	0.79 (95% CI 0.74 to 0.83)
Calibration intercept^b^	−0.08 (95% CI −0.98 to 0.52) (95% PrI −3.34 to 3.18)	0.02 (95% CI −0.21 to 0.24)
Calibration slope^a^	0.89 (95% CI 0.71 to 1.07) (95% PrI 0.35 to 1.42)	0.82 (95% CI 0.64 to 1.00)

^a^: a value of 1 is perfect, ^b^: a value of 0 is perfect. CI: confidence interval. PrI: prediction interval. The CI quantifies the precision of the average performance, while the PrI reflects the variance of the performance across regions in the leave-region-out cross-validation.

### Validation cohort

**Figure 2.**
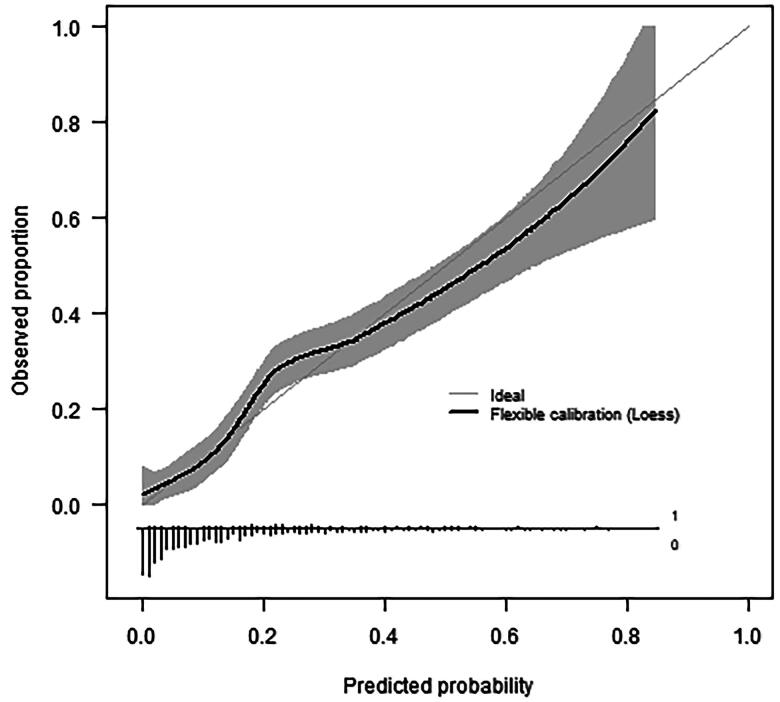
Flexible calibration curve in the temporal validation cohort (*n* = 830, 133 events) showing the predicted probability of hospital admission versus the observed proportion with hospital admission. The histogram on the horizontal axis shows the distribution of predicted risks among patients with (1, bars pointing upward) and without (0, bars pointing downward) hospital admission. Very short bars at predicted probabilities >0.5 indicate high predicted admission risks are rare.

The validation cohort consisted of 830 patients, with a similar age and gender distribution to the development cohort (median age 56, 51.8% female; Table 1a, Figure 1). Cough and dyspnoea were still the most prevalent symptoms at presentation, but more patients presented with non-specific symptoms such as myalgia and fatigue (Table 1b). The validation cohort appeared generally healthier than the development cohort concerning lifestyle and pre-existing comorbidities (Table 1a) but the frequency of hospital admissions (15.2%) and other adverse outcomes were higher (Table 2). The model performed slightly inferior in the second-wave temporal validation cohort compared to the first-wave geographic cross-validation but it was still able to estimate hospital admission risks accurately (Figure 2) and discriminate between those who would and would not be admitted within 14 days (C-index 0.79, Table 3).

During the validation period, 193 patients (23%) received one vaccination and 95 (11%) received two or more vaccinations. However, only 82 were vaccinated before their COVID-19-related GP visit, of which 15 (18%) were hospitalised. Fifty-five were vaccinated at least two weeks before their COVID-19 related GP visit, of which 7 (13%) were hospitalised. C-indexes were 0.76 and 0.72 in these subgroups. The average predicted hospitalisation risk was 19% in patients presenting at the GP at least 14 days after vaccination, while the observed risk was 13% (Table S9, Figure S10).

## Discussion

### Main findings

This study proposed a general practice prognostic model suitable to predict hospital admission due to COVID-19 within 14 days after a face-to-face GP consultation. The model included comorbidity and lifestyle variables, as well as current COVID-19-related symptoms, peripheral oxygen saturation, duration of complaints, body temperature and patient age and sex. The model had good discrimination (C-statistic 0.84) and calibration in the three regional cohorts. With a C-statistic of 0.79 and a calibration curve close to the diagonal, the model performed well in the second-wave temporal validation cohort despite considerable differences in overall hospitalisation risk, symptomatology, presence of comorbidities, dominant variants, and availability of tests and vaccination.

There were two unexpected findings. First, this study found that specific comorbidities (e.g. cancer and COPD) were negatively correlated with hospital admission after adjusting for vital signs and symptoms. The threshold for a face-to-face GP consultation may be lower for patients with severe comorbidities than for others, and hence, any correlation between comorbidity and COVID-19 prognosis may be weaker or reversed in the GP cohort compared to the general population. Second, second-wave patients presenting with COVID-19 to general practice appeared healthier but a higher percentage of patients were hospitalised compared to the first-wave development cohort. The increased availability of personal protective equipment (PPE) and knowledge of infection prevention may have lowered the threshold to physically assess healthier patients in general practice while the reduced strain on hospitals may have led to relatively more admissions. The hospitalisation rate in the first-wave cohort may also have been artificially lowered due to the inclusion of patients with other respiratory illnesses due to the unavailability of tests in primary care.

### Comparison with existing literature

The QCOVID models are the best-known prognostic models predicting hospital admission for COVID-19 [9,24]. They are intended for the general population (without COVID-19) instead of patients presenting to primary care with COVID-19-like symptoms or a positive test result. General population models predicting mortality are also available [9,25–28]. Our model targets a population of patients with symptoms presenting to their GP *via* a face-to-face consultation. The few models suitable for predicting the hospitalisation of patients with COVID-19 do not include symptoms or vital signs or are intended for remote assessment *via* digital platforms or mobile apps [7,8,10,11]. The reported c-statistic of the general population and primary care models to predict hospitalisation varied between 0.71 and 0.86. However, these performances cannot be compared directly to the c-statistics of 0.84 and 0.79 in the current analysis due to differences in studies populations and setting and type of validation.

### Strengths and limitations

This study met the a priori calculated sample size for model development, allowing for exploring a large set of relevant candidate predictors without inflating the risk of overfitting. The multicentre nature of this study increases the generalisability of the findings. However, some limitations need further discussion. First, the validation dataset predates the emergence of the omicron variant, so further validation is required. Any overestimation of hospitalisation risks could be remedied by recalibrating the model coefficients. We hypothesise the predictions conditional on vital signs and symptoms likely make our model less vulnerable to fluctuations in COVID-19 prevalence and perhaps also to changes in the severity of SARS-CoV-2 VOCs (variants of concern) and acquired immunity compared to the available models based on comorbidities alone. Second, the model does not take vaccination status into account. The vaccination campaign ran at full speed in the validation period but unfortunately, most vaccinated patients became infected before vaccination. Our estimates indicate the prediction model somewhat overestimated hospitalisation risks (19% versus 13%) in primary care patients with confirmed COVID-19 two to thirteen weeks after vaccination. However, we remain uncertain due to the small number of vaccinated patients in our dataset. Third, the use of routine care data from COVID-19 centres may have led to underreporting of comorbidities and selective reporting of symptoms. The temporal validation used patient files from regular general practice with more complete comorbidity and medication data. Fourth, the temporal validation data fell short of the target sample size but met the minimum criterion of 100 events for validation [29]. Fifth, the model was developed on patients with probable COVID-19, but the validation in patients with confirmed COVID-19 showed good predictive performance.

### Implications

In the past year (April 2022 to April 2023) there have been five COVID-19 waves in the Netherlands with up to 200 hospitalisations per day, compared to peaks of 300 hospitalisations per day earlier in the pandemic (October 2020 to April 2022) [30]. An updated version of our model could be built into software applications or online calculators to estimate hospitalisation risk after physical assessment by the GP, should COVID-19 cause a renewed strain on Dutch healthcare. It may aid in selecting high-risk COVID-19 patients that might benefit most from early interventions aimed to reduce complications and prevent hospital admission. However, to tackle changes over time due to novel SARS-CoV-2 variants, changing health policy and clinical practice, validating the model’s performance over time is critical. Model performance could be continuously or periodically checked by linking predicted risks based on GP data to hospital admission data. In any case, the current study demonstrates it is feasible to develop a risk prediction model to assist GPs in prognosis for an unknown emerging virus, should the need arise again.

## Conclusion

A model consisting of sex, smoking status, symptoms, vital signs, and comorbidities accurately estimated the risk of hospital admission due to COVID-19 in general practice, but further validation is needed to evaluate the accuracy of predicted hospitalisation risks in populations with acquired immunity and novel SARS-CoV-2 VOCs.

Box 1. Prediction model equation.

lp = -41.67 + 0.48 × man - 1.19 × smoker + 0.19 × auscultation abnormalities + 0.48 × confusion - 0.37 chest pressure or pain + 0.21 × cough - 0.50 × sputum + 0.55 × haemoptysis + 0.49 × stomach complaints (other than diarrhoea) + 0.23 × headache + 0.72 × chronic kidney disease - 1.25 × current treatment for malignancy - 0.64 × past treatment for malignancy - 0.53 × COPD + 0.06 × age (years) - 0.05 × age’ (years) + 1.11 × temperature (degrees Celsius) - 0.71 × temperature’ (degrees Celsius) - 0.06 × oxygen saturation (percent) - 0.15 × oxygen saturation’ (percent) + 0.30 × duration of complaints (days) - 0.34 × duration of complaints’ (days) probability of hospital admission within two weeks = $\frac{\text{exp}(\text{lp})}{1+\text{exp}(\text{lp})}$

Continuous variables are splines for which knots were placed at the 10%, 50% and 90% quantiles. For age: 37, 56 and 81; for temperature: 36.3, 37.1 and 38.4; for oxygen saturation: 92, 98 and 99; for duration of complaints: 0, 7 and 14. Lp = linear predictor

## Supplementary Material

Supplemental Material
