# Supplementary material for: Development and validation of a risk prediction model for hospital admission in COVID-19 patients presenting to primary care
Source: Eur J Gen Pract. 2024 Apr 29;30(1):2339488. doi: 10.1080/13814788.2024.2339488 (PMC11060008; doi:10.1080/13814788.2024.2339488)

Development and validation of a risk prediction model for hospital admission in COVID-19 patients presenting to primary care

SUPPORTIVE MATERIAL

Table of Contents

S1 Table. International Classification of Primary Care-2 Covid-19 Centre Urmond (Eastern South and Central Limburg)– First wave 2

S2 Table. International Classification of Primary Care-2 Covid-19 Centre Maastricht & Heuvelland (Western South Limburg) – First wave 4

S3 Table. International Classification of Primary Care-2 Covid-19 Centre Venlo/Venray (North Limburg)– First wave 7

S4 Appendix. Missing data and multiple imputation: extended information 10

S5 Table. Predictor effects 11

S6 Figure. Effects of continuous predictors on the predicted probability of hospital admission within 2 weeks, modelled with restricted cubic splines 12

S7 Figure. Distribution of predicted probabilities and calibration plots 13

S8 Figure. Forest plots of predictive performance within regions 15

S9 Table. Subgroup analysis by vaccination status in the temporal validation cohort 18

S10 Figure. Calibration plots in patients vaccinated prior to their first GP visit 18

S1 Table. International Classification of Primary Care-2 Covid-19 Centre Urmond (Eastern South and Central Limburg)– First wave

International Classification of Primary Care-2 (ICPC-2)	Codes	Process codes
A02.00	Koude rillingen	Chills
A03.00	Koorts	Fever
A04.00	Moeheid/zwakte	Weakness/tiredness general
A27.00	Angst voor andere ziekte	Fear of other disease NOS
A29.00	Andere algemene symptomen/klachten	General symptom/complaint other
A77.00	Andere virusziekte(n)	Viral disease other/NOS
A99.00	Andere gegeneraliseerde/niet gespecificeerde ziekte(n)	General disease NOS
D01.00	Gegeneraliseerde buikpijn/buikkrampen	Abdominal pain/cramps general
D06.00	Andere gelokaliseerde buikpijn	Abdominal pain localized other
D10.00	Braken	Vomiting
D11.00	Diarree	Diarrhoea
D73.00	Veronderstelde gastro-intestinale infectie	Gastroenteritis presumed infection
D88.00	Appendicitis	Appendicitis
K01.00	Pijn toegeschreven aan hart	Heart pain
K02.00	Druk/beklemming toegeschreven aan hart [ex. R02]	Pressure/tightness of heart
K04.00	Hartkloppingen/bewust van hartslag	Palpitations/awareness of heart
K05.00	Andere afwijkende/onregelmatige hartslag	Irregular heartbeat other
K70.00	Infectieziekte hartvaatstelsel	Infection of circulatory system
K74.00	Angina pectoris	Ischaemic heart disease with angina
K74.01	Instabiele angina pectoris	
K77.00	Decompensatio cordis	Heart failure
K77.01	Acute decompensatio cordis/astma cardiale	Acute heart failure
K77.02	Chronische decompensatio cordis	Heart failure chronic
K78.00	Boezemfibrilleren/-fladderen	Atrial fibrillation/flutter
K93.00	Longembolie/longinfarct	Pulmonary embolism
L04.00	Borstkas symptomen/klachten	Chest symptom/complaint
L18.00	Spierpijn	Muscle pain
L81.02	Ribcontusie	Rin contusion
N01.00	Hoofdpijn [ex. N02,N89,R09]	Headache
R01.00	Pijn toegeschreven aan luchtwegen [ex. R09]	Pain respiratory system
R02.00	Dyspnoe/benauwdheid toegeschreven aan luchtwegen [ex. K02]	Shortness of breath/dyspnoea
R03.00	Piepende ademhaling	Wheezing
R05.00	Hoesten	Cough
R21.00	Symptomen/klachten keel	Throat symptom/complaint

R21.01	Keelpijn	Sore throat
R22.00	Symptomen/klachten tonsillen	Tonsil’s symptom/complaint
R24.00	Haemoptoë	Haemoptysis
R27.00	Angst voor andere ziekte luchtwegen	Fear of respiratory disease, other
R29.02	Prikkelbare luchtwegen	Irritable respiratory tract
R74.00	Acute infectie bovenste luchtwegen	Upper respiratory infection acute
R74.01	Gewone verkoudheid	Common cold
R74.02	Acute pharyngitis	Acute pharyngitis
R75.00	Acute/chronische rhinosinusitis	Sinusitis acute/chronic
R75.01	Acute rhinosinusitis	Sinusitis acute
R75.02	Chronische rhinosinusitis	Sinusitis chronic
R76.00	Acute tonsillitis/peritonsillair abces	Tonsillitis acute/peritonsillar abscess
R76.01	Acute tonsillitis	Tonsillitis acute
R76.02	Peritonsillair abces	Peritonsillar abscess
R78.00	Acute bronchitis/bronchiolitis	Acute bronchitis/bronchiolitis
R80.00	Influenza [ex. R81]	Influenza
R81.00	Pneumonie	Pneumonia
R83.00	Andere infectie(s) luchtwegen	Respiratory infection other
R88.00	Ander letsel luchtwegen	Injury respiratory other
R95.00	Emfyseem/COPD	Chronic obstructive pulmonary disease
R96.00	Astma	Asthma
R96.01	Hyperreactiviteit luchtwegen	Hyper reactivity respiratory
R96.02	Allergisch astma	Allergic asthma
R97.00	Hooikoorts/allergische rhinitis	Allergic rhinitis
R98.00	Hyperventilatie	Hyperventilation syndrome
U70.00	Acute pyelonephritis/pyelitis	Pyelonephritis/pyelitis
U71.00	Cystitis/urineweginfectie	Cystitis/urinary infection other
U71.01	Cystitis	Cystitis

NOS = not otherwise specified

S2 Table. International Classification of Primary Care-2 Covid-19 Centre Maastricht & Heuvelland (Western South Limburg) – First wave

International Classification of Primary Care-2 (ICPC-2)	Titel	Title
A02.00	Koude rillingen	Chills
A03.00	Koorts	Fever
A04.00	Moeheid/zwakte	Weakness/tiredness general
A05.00	Algehele achteruitgang	Feeling ill
A27.00	Angst voor andere ziekte	Fear of other disease NOS
A29.00	Andere algemene symptomen/klachten	General symptom/complaint other
A76.00	Andere virusziekte met exantheem	Viral exanthem other
A77.00	Andere virusziekte(n)	Viral disease other/NOS
A78.00	Andere infectieziekte(n)	Infectious disease other/NOS
A96.00	Dood/overlijden [ex. A95]	Death
A96.01	Natuurlijke dood	Death natural cause
A99.00	Andere gegeneraliseerde/niet gespecificeerde ziekte(n)	General disease NOS
D01.00	Gegeneraliseerde buikpijn/buikkrampen	Abdominal pain/cramps general
D06.00	Andere gelokaliseerde buikpijn	Abdominal pain localized other
D10.00	Braken	Vomiting
D11.00	Diarree	Diarrhoea
D73.00	Veronderstelde gastro-intestinale infectie	Gastroenteritis presumed infection
D88.00	Appendicitis	Appendicitis
K01.00	Pijn toegeschreven aan hart	Heart pain
K02.00	Druk/beklemming toegeschreven aan hart [ex. R02]	Pressure/tightness of heart
K04.00	Hartkloppingen/bewust van hartslag	Palpitations/awareness of heart
K05.00	Andere afwijkende/onregelmatige hartslag	Irregular heartbeat other
K70.00	Infectieziekte hartvaatstelsel	Infection of circulatory system
K74.00	Angina pectoris	Ischaemic heart disease with angina
K74.01	Instabiele angina pectoris	Ischaemic heart disease with instable angina
K75.00	Acuut myocardinfarct	Acute myocardial infarction
K77.00	Decompensatio cordis	Heart failure
K77.01	Acute decompensatio cordis/astma cardiale	Acute heart failure
K77.02	Chronische decompensatio cordis	Heart failure chronic
K78.00	Boezemfibrilleren/-fladderen	Atrial fibrillation/flutter
K93.00	Longembolie/longinfarct	Pulmonary embolism
L04.00	Borstkas symptomen/klachten	Chest symptom/complaint
L18.00	Spierpijn	Muscle pain
L81.02	Ribcontusie	Rib contusion

L99.06	Syndroom van Tietze	Tietze syndrome
N01.00	Hoofdpijn [ex. N02,N89,R09]	Headache
R01.00	Pijn toegeschreven aan luchtwegen [ex. R09]	Pain respiratory system
R02.00	Dyspnoe/benauwdheid toegeschreven aan luchtwegen [ex. K02]	Shortness of breath/dyspnoea
R03.00	Piepende ademhaling	Wheezing
R04.00	Andere problemen ademhaling	Cough
R05.00	Hoesten	Headache
R07.00	Niezen/neusverstopping/loopneus	Sneezing/nasal congestion
R21.00	Symptomen/klachten keel	Throat symptom/complaint
R21.01	Keelpijn	Sore throat
R22.00	Symptomen/klachten tonsillen	Tonsil's symptom/complaint
R23.00	Symptomen/klachten stem	Voice symptom/complaint
R24.00	Haemoptoë	Haemoptysis
R27.00	Angst voor andere ziekte luchtwegen	Fear of respiratory disease, other
R29.02	Prikkelbare luchtwegen	Irritable respiratory tract
R72.01	Streptokokken-angina	Strep throat
R74.00	Acute infectie bovenste luchtwegen	Upper respiratory infection acute
R74.01	Gewone verkoudheid	Common cold
R74.02	Acute pharyngitis	Acute pharyngitis
R75.00	Acute/chronische rhinosinusitis	Sinusitis acute/chronic
R75.01	Acute rhinosinusitis	Sinusitis acute
R75.02	Chronische rhinosinusitis	Sinusitis chronic
R76.00	Acute tonsillitis/peritonsillair abces	Tonsillitis acute/peritonsillar abscess
R76.01	Acute tonsillitis	Tonsillitis acute
R76.02	Peritonsillair abces	Peritonsillar abscess
R77.00	Acute laryngitis/tracheïtis	Laryngitis/tracheitis acute
R78.00	Acute bronchitis/bronchiolitis	Acute bronchitis/bronchiolitis
R80.00	Influenza [ex. R81]	Influenza
R81.00	Pneumonie	Pneumonia
R83.00	Andere infectie(s) luchtwegen	Respiratory infection other
R84.00	Maligniteit bronchus/long	Malignant neoplasm bronchus/lung
R88.00	Ander letsel luchtwegen	Injury respiratory other
R90.00	Hypertrofie/chronische infectie tonsillen/adenoïd	Hypertrophy tonsils/adenoids
R91.00	Chronische bronchitis/bronchiëctasieën	Bronchitis chronic/bronchiectasis
R91.01	Chronische bronchitis	Bronchitis chronic
R91.02	Bronchiëctasieën	Bronchiectasis
R95.00	Emfyseem/COPD	Chronic obstructive pulmonary disease
R96.00	Astma	Asthma
R96.02	Allergisch astma	Allergic asthma
R97.00	Hooikoorts/allergische rhinitis	Allergic rhinitis
R98.00	Hyperventilatie	Hyperventilation syndrome

R99.00	Andere ziekte(n) luchtwegen	Respiratory disease other
U70.00	Acute pyelonephritis/pyelitis	Pyelonephritis/pyelitis
U71.00	Cystitis/urineweginfectie	Cystitis/urinary infection other
U71.01	Cystitis	Cystitis
U71.02	Urineweginfectie neg [ex. venerisch]	Acute/chronic cystitis (non-venereal)

S3 Table. International Classification of Primary Care-2 Covid-19 Centre Venlo/Venray (North Limburg)– First wave

International Classification of Primary Care-2 (ICPC-2)	Titel	Title
A02.00	Koude rillingen	Chills
A03.00	Koorts	Fever
A04.00	Moeheid/zwakte	Weakness/tiredness general
A05.00	Algehele achteruitgang	Feeling ill
A27.00	Angst voor andere ziekte	Fear of other disease NOS
A29.00	Andere algemene symptomen/klachten	General symptom/complaint other
A76.00	Andere virusziekte met exantheem	Viral exanthem other
A77.00	Andere virusziekten	Viral disease other/NOS
A78.00	Andere infectieziekten	Infectious disease other/NOS
A96.00	Dood/overlijden [ex. A95]	Death
A96.01	Natuurlijke dood	Death natural cause
A99.00	Andere gegeneraliseerde/niet gespecificeerde ziekten	General disease NOS
D01.00	Gegeneraliseerde buikpijn/buikkrampen	Abdominal pain/cramps general
D06.00	Andere gelokaliseerde buikpijn	Abdominal pain localized other
D10.00	Braken	Vomiting
D11.00	Diarree	Diarrhoea
D73.00	Veronderstelde gastro-intestinale infectie	Gastroenteritis presumed infection
D88.00	Appendicitis	Appendicitis
K01.00	Pijn toegeschreven aan hart	Heart pain
K02.00	Druk/beklemming toegeschreven aan hart [ex. R02]	Pressure/tightness of heart
K04.00	Hartkloppingen/bewust van hartslag	Palpitations/awareness of heart
K05.00	Andere afwijkende/onregelmatige hartslag	Irregular heartbeat other
K74.00	Angina pectoris	Ischaemic heart disease with angina
K74.01	Instabiele angina pectoris	Ischaemic heart disease with instable angina
K75.00	Acuut myocardinfarct	Acute myocardial infarction
K77.00	Decompensatio cordis	Heart failure
K77.01	Acute decompensatio cordis/astma cardiale	Acute heart failure
K78.00	Boezemfibrilleren/-fladderen	Atrial fibrillation/flutter
K93.00	Longembolie/longinfarct	Pulmonary embolism
L04.00	Borstkas symptomen/klachten	Chest symptom/complaint
L18.00	Spierpijn	Muscle pain
L81.02	Ribcontusie	Rib contusion
L99.06	Syndroom van Tietze	Tietze syndrome

N01.00	Hoofdpijn [ex. N02,N89,R09]	Headache
R02.00	Dyspnoe/benauwdheid toegeschreven aan luchtwegen [ex. K02]	Pain respiratory system
R03.00	Piepende ademhaling	Shortness of breath/dyspnoea
R04.00	Andere problemen ademhaling	Wheezing
R05.00	Hoesten	Cough
R21.00	Symptomen/klachten keel	Throat symptom/complaint
R21.01	Keelpijn	Sore throat
R22.00	Symptomen/klachten tonsillen	Tonsil's symptom/complaint
R24.00	Haemoptoë	Haemoptysis
R25.00	Abnormaal sputum/slijm	Sputum/phlegm abnormal
R27.00	Angst voor andere ziekte luchtwegen	Fear of respiratory disease, other
R29.00	Andere symptomen/klachten luchtwegen	Respiratory symptom/complaint other
R29.02	Prikkelbare luchtwegen	Irritable respiratory tract
R74.00	Acute infectie bovenste luchtwegen	Upper respiratory infection acute
R74.01	Gewone verkoudheid	Common cold
R74.02	Acute pharyngitis	Acute pharyngitis
R75.00	Acute/chronische sinusitis	Sinusitis acute/chronic
R75.01	Acute sinusitis	Sinusitis acute
R76.00	Acute tonsillitis/peritonsillair abces	Tonsillitis acute/peritonsillar abscess
R76.01	Acute tonsillitis	Tonsillitis acute
R76.02	Peritonsillair abces	Peritonsillar abscess
R77.00	Acute laryngitis/tracheïtis	Laryngitis/tracheitis acute
R78.00	Acute bronchitis/bronchiolitis	Acute bronchitis/bronchiolitis
R80.00	Influenza [ex. R81]	Influenza
R81.00	Pneumonie	Pneumonia
R83.00	Andere infecties luchtwegen	Respiratory infection other
R84.00	Maligniteit bronchus/long	Malignant neoplasm bronchus/lung
R88.00	Ander letsel luchtwegen	Injury respiratory other
R95.00	Emfyseem/COPD	Chronic obstructive pulmonary disease
R96.00	Astma	Asthma
R96.02	Allergisch astma	Allergic asthma
R97.00	Hooikoorts/allergische rhinitis	Allergic rhinitis
R98.00	Hyperventilatie	Hyperventilation syndrome
R99.00	Andere ziekten luchtwegen	Respiratory disease other
U70.00	Acute pyelonephritis/pyelitis	Pyelonephritis/pyelitis
U71.00	Cystitis/urineweginfectie	Cystitis/urinary infection other
U71.01	Cystitis	Cystitis

Note: The selected ICPC codes in the validation cohort were R83.03 SARS-CoV-2 in addition to any other ICPC codes that may indicate COVID-19 (see S1 Table, S2 Table, S3 Table).

S4 Appendix. Missing data and multiple imputation: extended information

Our data did not allow a distinction between a patient not suffering from a comorbidity or not having certain symptoms and missing comorbidity/symptoms data. Hence, if a comorbidity (or medication indicating disease) or symptom was not in the patient's file, it was assumed the patient did not have that comorbidity or symptom. The same approach was used for smoking and the patient being overweight. If COVID-19-status was unknown, it was assumed the patient was not tested. In 54% of patients, the exact number of days since the onset of complaints was not reported in the patient files, but the reported duration of complaints could be mapped on a categorical variable (since today, since 1-4 days, since 5-9 days, since >9 days). For these patients, category midpoints were used, using 14 days if duration of complaints was >9 days based on the median value in similar patients that reported an exact duration >9 days.

The variables with the highest proportions of missing values were CRP (81% missing), exact body temperature measured at home (55%), exact duration of complaints (54%), respiratory rate (45%), systolic and diastolic blood pressure (44% each), heart rate (15%), exact body temperature measured at the GP (13%) and oxygen saturation (9%). Other variables had max. 1% missings. CRP and exact body temperature measured at home were excluded from analysis due to the high proportion of missingness. Other variables were multiply imputed (100 imputations).

All candidate predictors and additional demographic data, comorbidity data, symptoms, and vital functions from the first two GP visits, and hospital data were considered as independent variables in the imputation models. Constant and collinear variables were excluded as independent variables, as well as variables with an outflux < 0.5 (which indicates little potential usefulness for imputing other variables). Per variable to imputed, the imputation model was further tweaked using the mice quickpred function by including independent variables that had a correlation with the variable to be imputed or the missingness status of the variable to be imputed of at least 0.1, and excluding variables that had less than 5% usable cases (i.e. <5% non-missing independent variable data when the dependent variable was missing). Age and hospital admission within 14 days of the first GP visit were always included in the imputation models as independent variables, for all imputed variables. This resulted in rich imputation models with a median number of 22 independent variables per imputation model. The methods used for the imputation models were predictive mean matching for continuous variables, logistic regression for binary variables, and multinomial logit models for categorical variables. If the patient file indicated the patient had fever during the GP visit, the imputed body temperature was restricted to be minimally 38 degrees Celsius, whereas it was restricted to be maximally 37 degrees Celsius if the patient file indicated the patient did not have a fever. The maximum number of iterations was ten and convergence was checked using convergence plots.

Imputations were done separately for each region and separately for development and validation data to avoid 'leakage'. We used the psfmi_lr function in the psfmi R-package with the "D1" method for backward variable selection to perform model building on the imputed data within each split of the cross-validation procedure. This performs backward selection on the pooled model, using the complete variance-covariance matrix to accommodate restricted cubic splines.

S5 Table. Predictor effects

Predictor	OR	95% CI OR lower limit	95% CI OR upper limit
(Intercept)	/	/	/
Man	1.70	1.29	2.23
Current smoker	0.27	0.09	0.78
Auscultation abnormalities	1.24	0.92	1.66
Confusion	1.7	1.05	2.76
Chest pressure or pain	0.67	0.46	0.96
Cough	1.26	0.92	1.73
Sputum	0.57	0.38	0.86
Haemoptysis	1.85	0.8	4.27
Stomach complaints (excl. diarrhoea)	1.72	1.31	2.26
Headache	1.29	0.91	1.83
Chronic kidney disease	2.23	1.25	4
Current treatment for malignancy	0.25	0.07	0.85
Past treatment for malignancy	0.49	0.24	1
COPD	0.55	0.36	0.85
Age	1.08	1.04	1.11
Age’	0.95	0.93	0.98
Body temperature	3.41	1.65	7.08
Body temperature’	0.46	0.19	1.09
Oxygen saturation	0.93	0.86	1
Oxygen saturation’	0.85	0.77	0.93
Duration of complaints	1.39	1.28	1.52
Duration of complaints’	0.69	0.62	0.76

’ Spline transformation. Restricted cubic splines are a way to represent a relationship between two variables (like age and logit of hospitalisation). Instead of assuming a straight line for the relation between the predictor and logit of the outcome, for the entire range of predictor values, a spline uses smaller sections defined by the predictor values to fit non-linear curves that join together smoothly. A knot is a specific point where these sections connect. Here, continuous variables are splines for which knots were placed at the 10%, 50% and 90% quantiles. For age: 37, 56 and 81, for temperature: 36.3, 37.1 and 38.4, for oxygen saturation: 92, 98 and 99, for duration of complaints: 0, 7 and 14. The transformation denoted in the table as predictor’ can be calculated in R using the rcspline.eval function in the rms package. For example, Age’could be calculated as Validationdata$Age_accent <- rcspline.eval(Validationdata$Age, knots=c(37,56,81)).
Lp = linear predictor

S6 Figure. Effects of continuous predictors on the predicted probability of hospital admission within 2 weeks, modelled with restricted cubic splines

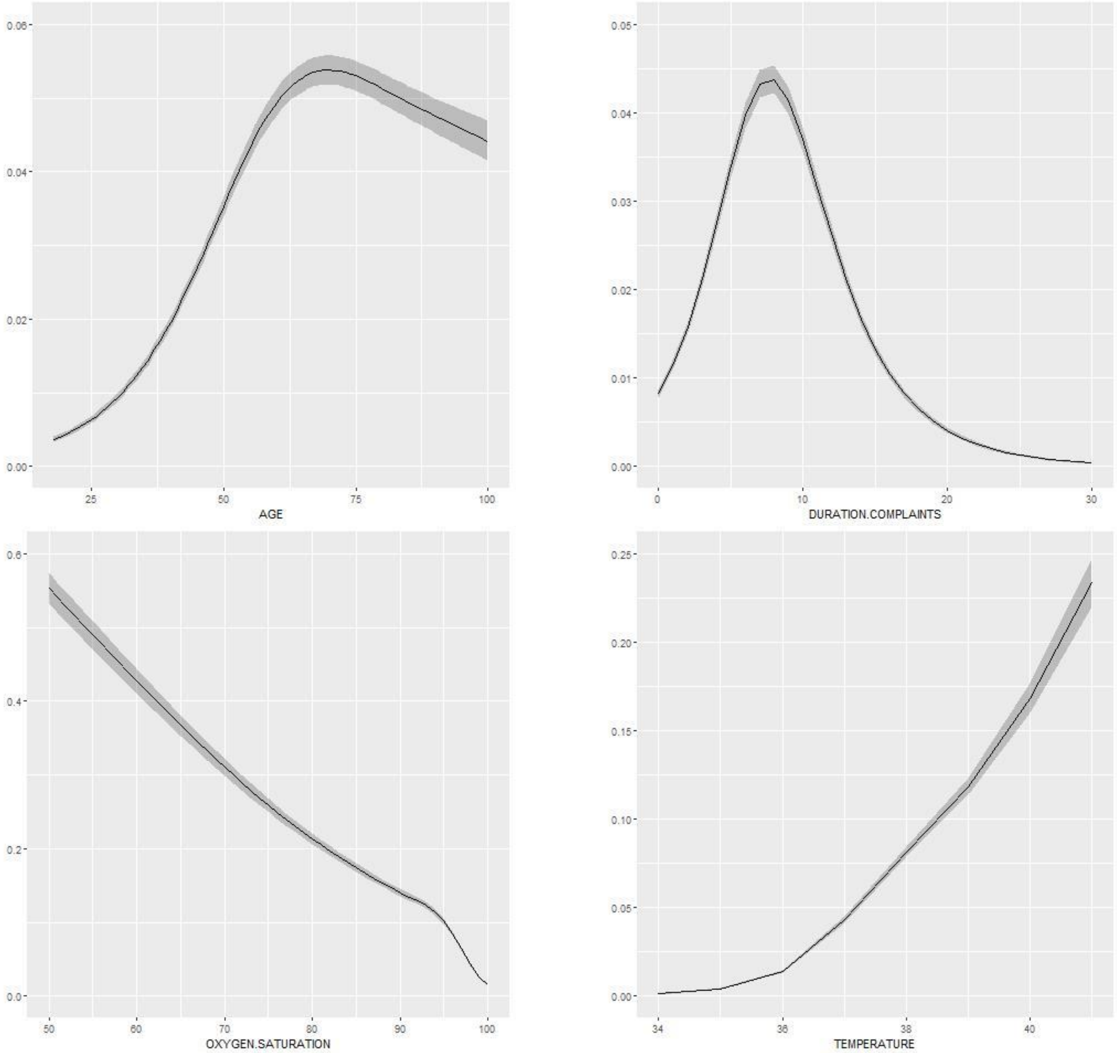

The vertical axis is the probability of hospital admission on a scale of 0 to 1. The predicted probabilities are shown for a reference patient of 55, complaints for 7 days, with a body temperature of 37 degrees Celsius, oxygen saturation of 98, who is a non-smoker, without auscultation abnormalities, confusion, chest pain or pressure, sputum, haemoptysis, stomach complaints, headache, chronic kidney disease, current or past treatment for malignancy, or COPD. From top left to bottom right: Age (in years), duration of complaints (in days), oxygen saturation (in percent), body temperature (in Degrees Celsius).

S7 Figure. Distribution of predicted probabilities and calibration plots

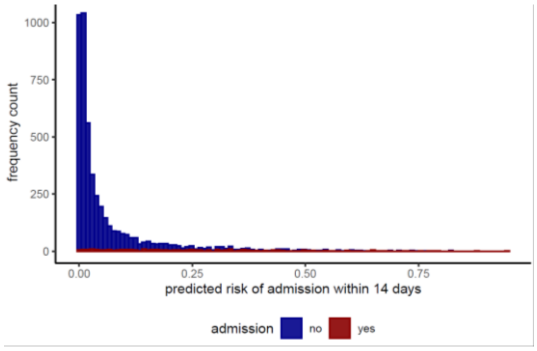

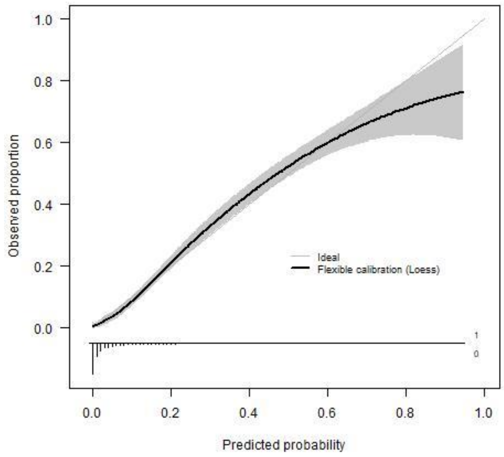

Development cohort (apparent)

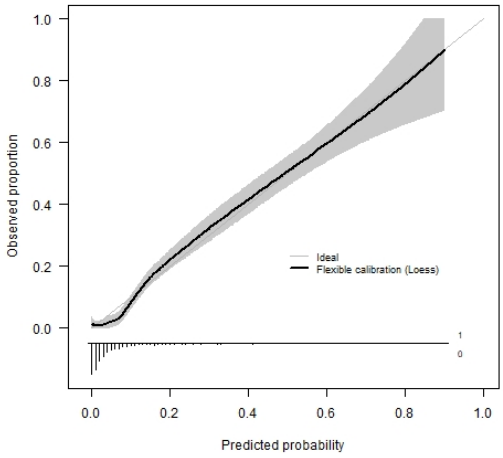

Western South Limburg – leave region out cross-validation

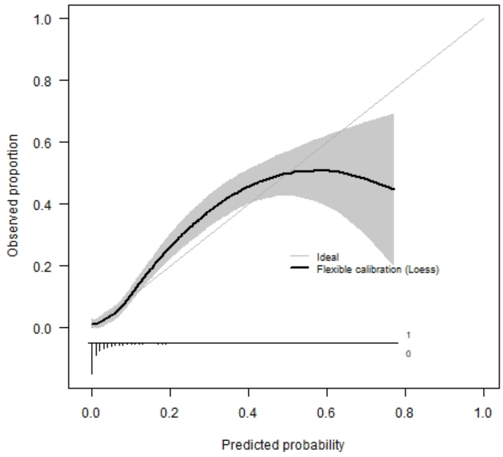

Eastern South & Central Limburg – leave region out cross-validation

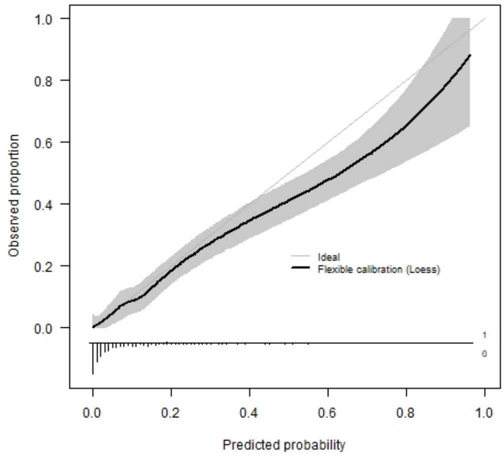

North Limburg – leave region out cross-validation

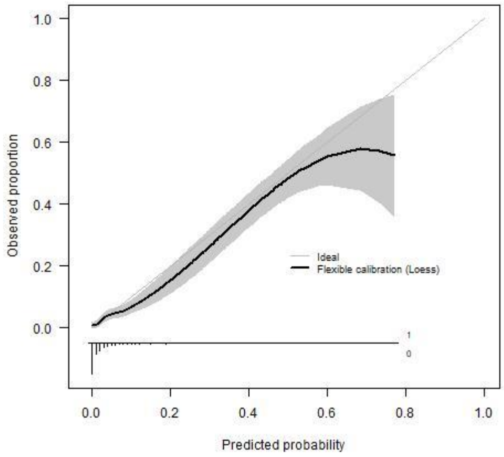

Women – subgroup analysis

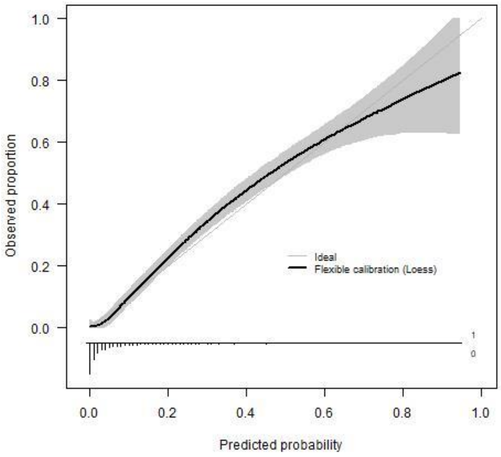

Men – subgroup analysis

S8 Figure. Forest plots of predictive performance within regions

A. C-index

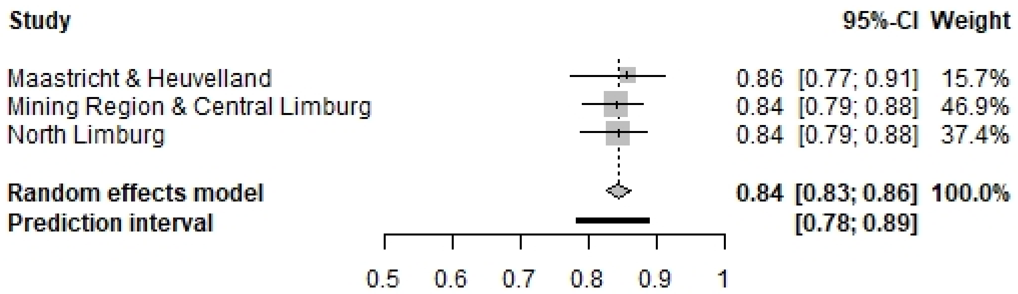

Footnote: Study codes stand for regions: Maastricht & Heuvelland: Western South Limburg; Mining Region & Central Limburg: Eastern South Limburg; North Limburg: North Limburg.

B. Calibration intercept

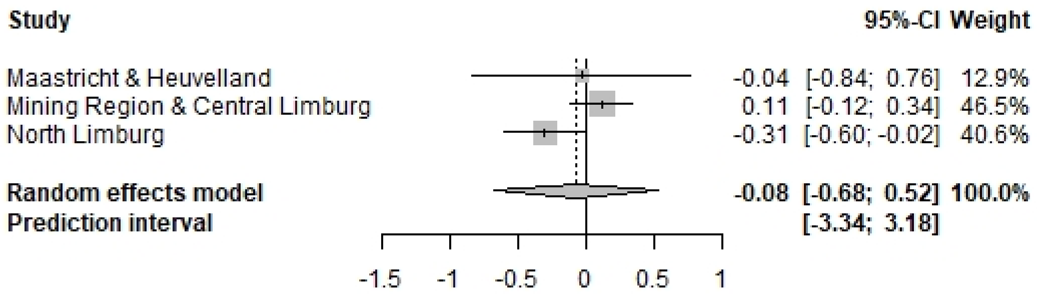

Footnote: Study codes stand for regions: Maastricht & Heuvelland: Western South Limburg; Mining Region & Central Limburg: Eastern South Limburg; North Limburg: North Limburg.

C. Calibration slope

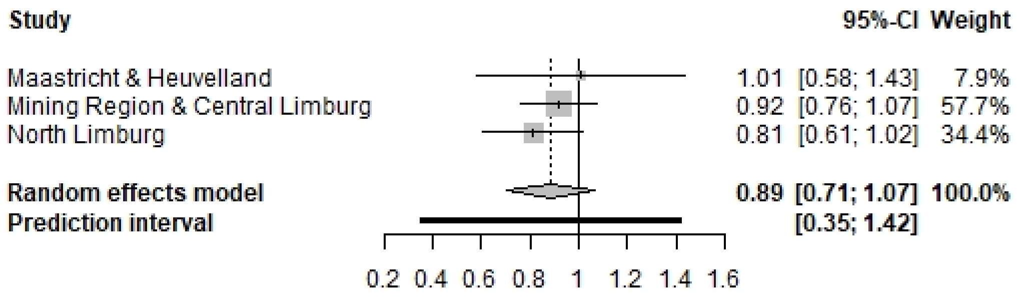

Footnote: Study codes stand for regions: Maastricht & Heuvelland: Western South Limburg; Mining Region & Central Limburg: Eastern South Limburg; North Limburg: North Limburg.

S9 Table. Subgroup analysis by vaccination status in the temporal validation cohort

	At least 1 vaccine at least 14 days prior to first visit (n=55)	At least 1 vaccine prior to first visit (n=82)
**C-index[a]**	0.76 (95% CI 0.51 to 0.91)	0.72 (95% CI 0.54 to 0.85)
**Calibration intercept[b]**	-0.59 (95% CI -1.49 to 0.31)	-0.10 (95%CI -0.74 to 0.55)
**Calibration slope[a]**	0.82 (95% CI 0.05 to 1.58)	0.63 (95% CI 0.13 to 1.13)
**Average predicted risk versus observed hospitalization risk**	19% versus 13%	19% versus 18%
	**Not vaccinated at least 14 days prior to first visit**	**Not vaccinated prior to first visit**
**C-index[a]**	0.79 (95% CI 0.75 to 0.83)	0.80 (95% CI 0.75 to 0.84)
**Calibration intercept[b]**	0.06 (95% CI -0.17 to 0.29)	0.03 (95% CI -0.21 to 0.27)
**Calibration slope[a]**	0.83 (95% CI 0.64 to 1.01)	0.84 (95% CI 0.65 to 1.03)
**Average predicted risk versus observed hospitalization risk**	15% versus 16%	15% versus 16%

a: a value of 1 is perfect, b: a value of 0 is perfect.

S10 Figure. Calibration plots in patients vaccinated prior to their first GP visit

Vaccinated at least 14 days prior to the first visit (n=55, 7 events)

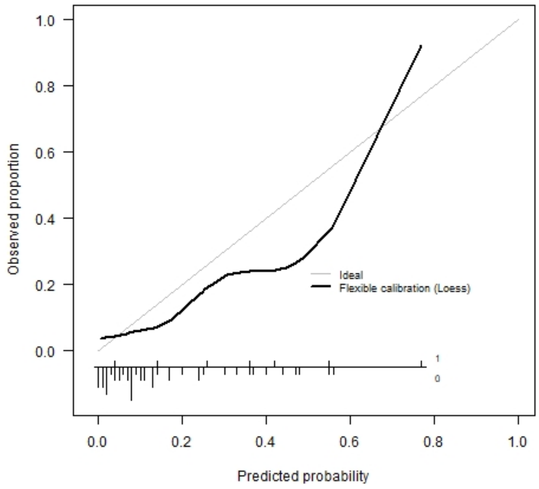

Vaccinated prior to the first visit (n=82, 15 events)

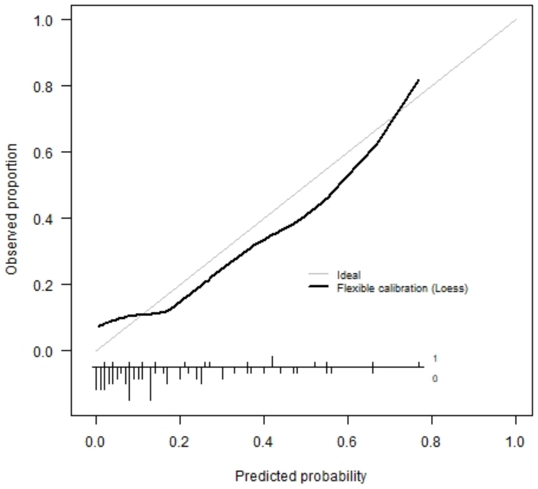

Supplement: Supplemental Material [file IGEN_A_2339488_SM8791.zip › ejgp-2023-0097-File004.pdf]
